# Supplementary material for: Kangaroo mother care prior to clinical stabilisation: Implementation barriers and facilitators reported by caregivers and healthcare providers in Uganda
Source: PLOS Glob Public Health. 2024 Jul 31;4(7):e0002856. doi: 10.1371/journal.pgph.0002856 (PMC11290675; doi:10.1371/journal.pgph.0002856)
Supplement: S1 File — (DOCX) [file pgph.0002856.s002.docx]

**DATA SUMMARY**

**Barriers & facilitators of KMC before stabilization from Healthcare workers’ perspective**.

**Facilitators of KMC**

From the interview with the healthcare workers on the facilitators and barriers of practicing KMC prior to stabilization, the emerging themes under facilitators of KMC evolved around the 5 WHO building blocks. That is infrastructure, medicines and supplies, human resources for health, leadership and family involvement/support.

**1.1 Adequate spaces and KMC beds (infrastructure)**

Having adequate spaces for KMC beds where the mothers could do KMC in privacy was reported as one of the things that would facilitate KMC at the different hospitals as illustrated in excerpt below:

| **Sex** | **Age** | **Quotes** | **Comments** |
| --- | --- | --- | --- |
|  |  | *“The facilitation comes in as the availability of the enough and comfortable beds, the availability of the concentrators to provide oxygen that is required and the space. Sometimes we have two or three mothers performing Kangaroo Mother Care. Sometimes, they can have the babies and keep them; with enough space available” (Nurse – AH).* | Lack of medical supplies  AND  Infrastructure |
|  |  | *“We have to have beds for such mothers. We have to have a ward for Kangaroo; they should know that the preterms are in this ward of Kangaroo and the beds are there for the mothers. NICU babies don’t have beds and the only Kangaroo that is done there is on the chairs when they go to breast feed the babies. After feeding, they put the babies in Kangaroo. They don’t get to practice Kangaroo well, they just put the baby there for 30 minutes and they chase them from NICU. Then they will come back after four hours. But, if they knew that this was for preterms and the Kangaroo is theirs, and they cater for them when the mothers are there. Actually, we need beds” (Nurse- CH).* | Infrastructure |

**1.2 Sub-theme: Availability of medicines, supplies and equipment**

Availability of medicines and supplies to care for small and sick newborns was mentioned as a facilitator of KMC prior to stability as caregivers will develop hope of their newborns’ survival and spend more time doing KMC than moving out of the hospital to buy medicines.

| **Sex** | **Age** | **Quote** | **Comment** |
| --- | --- | --- | --- |
| **F** |  | *“we also need functional monitors because we need to monitor these babies’ circulation and we see how they are doing when they are in KMC. So that will greatly help us” (nurse - CH).* | Medicines and supplies |
| **M** |  | *“The facilitation comes in as the availability of the enough and comfortable beds, the availability of the concentrators to provide oxygen that is required and the space. Sometimes we have two or three mothers performing Kangaroo Mother Care. Sometimes, they can have the babies and keep them; with enough space available” (Nurse – AH).* |  |

**1.3 Sub-theme: Adequate staffing (human resources for health)**

Having enough trained healthcare workers who are able to handle small and sick newborns was reported as an important facilitator of KMC prior to stabilisation. This will improve on the care of preterm newborns, and counselling/education of caregivers in doing KMC.

| **Sex** | **age** | **Quotes** | **Comments** |
| --- | --- | --- | --- |
|  |  | *“Actually, the first thing should be more nurses; the nurses are very few. Also educating the nurses because not all of them know how to take care of the neonates; some don’t know how to put up a CPAP. So, they should train them” (Nurse- CH).* | Staffing levels.  Training of HCWs |

**1.4 Caregiver health education on KMC (leadership/governance)**

There was a general lack of knowledge about KMC among mothers of the unstable babies and the health care workers felt that creating awareness through health education would help facilitate the implementation of KMC.

| **Sex** | **Age** | **Quote** | **comment** |
| --- | --- | --- | --- |
|  |  | *“There is ignorance; there is not enough education about what Kangaroo is. They just don’t know it is skin to skin. You find that she is dressed and also the baby is dressed: and then she puts the baby in the chest and thinks that is Kangaroo. Then you explain to her that it is skin to skin; and tell her to remove the blouse. So enough education is required on that” (Respondent 1- CH).* | Lack of knowledge about KMC for the mothers.-Barrier  Need for health education. -governance |
|  |  | *“In my opinion, if I am to rate administrative involvement, it is almost 20% support and that is the provision of those four beds (interrupted by one respondent) and the oxygen which is in most cases running out every day and you have to chase (follow up) people for oxygen. So, we actually need people to push them to understand kmc so that they can help us. But it is not something that they have put to the front that it needs attention” (respondent 3 - AH).* | Hospital leadership involvement |

**1.5 Social Support for the mothers (Family involvement and support)**

Provision of basic needs like food would allow mothers to stay longer in hospitals and be able to practice KMC.

| **Sex** | **Age** | **Quote** | **comments** |
| --- | --- | --- | --- |
|  |  | *“KMC for unstable babies and preterm babies requires some time in the hospital. If there would be any way that these mothers are provided with the basics like food and tea to supplement on what they can afford, we would see more babies survive. There are people you may not sympathise with what they are saying. Someone will tell you I don’t have what to eat and actually the person has nothing to eat. So if there are ways of seeing how they provide for the legal basic for these people who are going to stay longer because their babies have not stabilised, it would help improve” (Nurse – DH).* | **Family support** |

**Barriers of KMC prior to stabilization**

**1.0 Theme: Personal**

**1.1 Sub-theme: Fear of deterioration in health status:** Health care workers were concerned that the condition of the newborn might deteriorate while in KMC given that the baby is still unstable. The fear was associated with lack of new training in KMC before stabilisation given that they are familiar with KMC after stabilisation. They also reported that, the mothers are afraid of accidental suffocation of the newborns and disconnection of the oxygen while in KMC.

| **Sex** | **Age** | **Quotes** | **Comments** |
| --- | --- | --- | --- |
| F | - | *“The fear in the health workers is that we have been used to the analog state I would say where we used to handle preterm babies in an incubator or a radiant warmer and the unstable for example on oxygen and yours would be looking at the baby in an incubator or radiant warmer and they are on oxygen until they stabilise that is when we would initiate KMC. But here comes OMWANA study which is telling us that even when the baby is unstable, you have to put them in KMC. We are still used to the other initial training where we had a baby in an incubator on oxygen slowly being weaned off and then put in KMC. So, I think that is where the fear comes from” (Respondent 5,enrolled nurse – DH).* | - Fear of deterioration in health status. - Lack of knowledge in KMC before stabilization |
| M |  | *“initially to start KMC for very unstable babies is caring for the mothers and the health workers because this baby could still be on a drip, oxygen and they are very small so the mother would get scared about how to put this baby in the chest and the baby improves. Even the health care workers when the baby is still on oxygen, we are a bit anxious” (Respondent 3 – Medical officer – DH)* | Fear of deterioration |
|  |  | *“Practicing KMC is scary to the mother first of all because this is a small sick baby who is not doing well. Putting this baby in KMC is really scary because the mother may have fear of suffocating the baby, wondering if she will do it or afford to maintain what the health worker is doing. So, it is scary to the mother” (Respondent 7, senior nursing officer - DH)* |  |
|  |  | *“Sometimes the mothers can say that my baby will die. They do not know that putting the unstable baby in the chest can help them by enabling the baby to survive. They think that the baby will die and that putting the baby in the incubator is what will save the baby” (Respondent 1- Midwife – DH)*. | Fear of suffocating the baby.  Incubator is superior to KMC |
|  |  | *“Maybe the other fear to the mother is about the oxygen. When you put the unstable baby in KMC with the oxygen prongs in, they think that the prongs will go off and the babies will die” (Respondent 6 - Midwife - DH).* | Fear of disconnecting oxygen prongs |

**1.2 sub-theme: Lack of adequate knowledge in KMC before stabilization.** The lack of experience and adequate knowledge about KMC in unstable preterm newborns created fear among healthcare workers that it may lead to deterioration of the newborn.

| **Sex** | **Age** | **Quotes** | **Comments** |
| --- | --- | --- | --- |
|  |  | *It is first of all something scary to have an unstable baby who is for example on oxygen and their circulations are a little fluctuating below 90 yet you would love to put them in KMC. If you have never done it, you feel like you might end up killing this baby if you are not doing the right thing” (Respondent 5, enrolled nurse- DH).* |  |
| **-** |  | *“In my opinion, the other fear is lack of knowledge and skills gap of doing KMC. For example, it was yesterday when we received a referral from a health centre. We got a preterm in that they pretended to put the baby in KMC but instead killed the baby because of the way they brought the baby with the whole face covered but not in KMC. So, I may say they lacked skills. That is another fear” (Respondent 6 – enrolled midwife –DH).* |  |

**1.3 Sub-theme: Maternal morbidity.** Mothers who deliver by caesarean Section cannot start doing KMC immediately and this will lead to delay in starting KMC.

| Sex | Age | Quotes | Comment |
| --- | --- | --- | --- |
| F | - | *Another challenge is; if a mother is a caesarean mother, you have enrolled and randomised her into kmc, she has to first be discharged from the post-natal ward and that is when she will start kmc yet kmc is supposed to be initiated immediately. So that is a challenge to us” (Respondent 1 – AH).* | Maternal morbidity |
|  |  | *“One is certainly where there is no one to do Kangaroo especially for a mother who has been operated upon and they don’t have a caretaker. They are still weak and still receiving medication. At times, it becomes a barrier” (Nurse- CH).* | Maternal morbidity |

**1.4 Sub-theme: Fear to handle the baby:** Young mothers tend to fear the newborn babies for the first days of delivery and this impedes the KMC prior to stability.

| Sex | Age | Quote | Comments |
| --- | --- | --- | --- |
|  |  | *“For some mothers mostly the young mothers that I have met fear the babies to the extent that when you bring the baby closer to her, even in a cloth, they fear the baby. Now putting the baby in the chest, she is just not used to the baby. I am not sure but maybe they fear to break. They just fear to handle the baby” (Respondent 2- DH).* |  |

**1.5 Discomfort and tiredness during KMC**

Physical discomfort and fatigue while doing KMC was reported as another barrier to KMC. The KMC position is uncomfortable and creates fatigue which creates difficulties in doing KMC for the mothers.

| Sex | Age | Quote | Comment |
| --- | --- | --- | --- |
| F |  | “*Fear of the mother putting the baby in the chest comes from wondering how to put the baby who is not breathing well in the chest and how to rest and sleep because most of the time, they are not used to the position used. Even if it is a health worker doing it, the position is not comfortable for them. So they have the fear that the baby will die in the chest” (Respondent 7 – senior nursing officer DH).* | Fear of harming the baby  Discomfort |
| F |  | *“Maybe I can talk of the discomfort. Sometimes it is tiresome for mothers who don’t have attendants. That is why sometimes you find that the baby is already cold because the mother has left the baby in the bed, and she is away. You talk to her and she says she doesn’t have attendants and gets tired of sleeping on the back for a long time. So sometimes I think it is tiresome if the mother has no attendants” (respondent 3 – AH).* | Tiresome for the mother |

**1.6 Lack of family-social support.**

Lack of a family member to help the caregiver while doing KMC was mentioned as one of the barriers of KMC prior to stabilization. Especially when the family believes that the newborn will not survive, they view KMC as a wastage of time and pull out the support.

| Sex | Age | Quotes | Comments |
| --- | --- | --- | --- |
| F |  | *“Looking at that small unstable baby, the fathers think that the health workers are wasting their time, this baby is going to die at any time, so they feel there is no benefit staying in the hospital. Most times, they abandon the mothers on ward because they feel it is a wastage of time since the baby is going to die” (Respondent 7- DH).* | Lack family support. |
|  |  | *“Some of the challenges; when mothers lack support from their care takers and husbands, some are also not willing to stay in the hospital, some are not willing to do KMC, some because of fear of the baby and fear of the condition of the baby. They also tend not to do it” (Respondent 2- DH).* | Lack of support |
|  |  | *“We have instances of fathers seeing the baby on oxygen and believes the baby is dead and some of disconnect and even disappear even after you have laboured to explain that the monitor shows that the baby’s circulation is 80-90 but doesn’t believe us and says we basawo want to keep them in the ward. But those who are a little literate can be explained to and they accept. I had a gentle man who stayed here for 3 months, and he was very good. He understood everything and, in the ward, we had him as a champion. He asked everyone to wash hands, leave shoes the other side, he knew how much milk is to be given every day and do everything. But I think most of it is about making those people understand how everything works and improve life” (Respondent 4 - DH).* | Lack of social support.  Lack knowledge on KMC |

**1.7 Other family obligations**. The lack of support also prompts mothers to opt for early discharge from hospital which is a barrier to KMC for unstable babies. Mothers without social and economic support and those who had other young children who were left uncared for at home found it hard to remain in hospital for longer periods of time.

| Age | Sex | Quote | Comments |
| --- | --- | --- | --- |
|  |  | *“They want to leave early because the of their financial status; they do not want to be in hospital longer, they have left other young one’s home. And others say, am not sure though, that their men will be taken so they need to go back home” (Respondent E, BH).* |  |
|  |  | *“The challenges to the mothers are due to socioeconomic status like resp3 has said, caring for a small baby takes a lot of time in the facility so this couple looks at home, they are renting, they have no jobs, they have other children at home. Now who is going to keep the children at home? It’s the mother, the father goes to look for money to support themselves. So, minus the mother at home, who is going to care for the children there? So, they insist that they want to go home because they are looking at this small baby who is going to die at any time, probably should not waste their time in the hospital because the father has to go and look for money to support themselves” (Respondent 7- DH).* |  |

**1.8 Sub-theme: Financial constraints**

Lack of financial support to meet the demands of hospital stay leads to caregivers’ demand for early discharge from the hospital thus discontinuing KMC.

| Age | Sex | Quote | Comments |
| --- | --- | --- | --- |
|  |  | *“We have a challenge of initiating kmc when the mother has no clothes at all. She only has herself. So you cannot tell her to do kmc because even if you give her the kmc wrap, it will not be enough. So, you end up leaving the baby in the radiant warmer than bringing her to kmc” (Respondent 1- AH).* |  |
|  |  | *“To add to what the rest have said, some of the fathers especially those with very low education link giving birth to a premature baby as a kind of bad omen from the woman. They sometimes think she is the problem they don’t know the causes as to why the baby can be born preterm, so they tend to reject the mother and the child. So that is another fear from it. The illiterate husbands think that the problem is the woman, witchcraft or she has a bad omen. So, it is a challenge” (Respondent 3- DH).* |  |
|  |  | *“We happen to work in a community where some people are not better off economically and yet for these babies, they may need to stay in the hospital for more days. So, when it comes to feeding, some men find it hard to stay here for 5, 7 or more days. We have seen men who have said I want my baby back home even when they are still on oxygen, and they are serious and say they cannot sustain them in the hospital. I think last week I had one, we tried counselling the mother with L and in fact I had talked to them and going to consent them the following day but in the night, I even called the gentleman on the phone and explained that you want this baby at home but we cannot discharge because the baby is on oxygen and might not reach home. The man insisted that he wanted the baby home because he couldn’t sustain them in the hospital” (respondent 4- DH).* |  |

**2.0 Theme: Institutional factors**

**2.1 Sub-theme: Lack of medicines and supplies:** Inadequate medicines and supplies to care for small and sick newborns hinders the continuity of KMC as mothers have to be sent to buy these supplies or babies have to be moved where they can be connected to oxygen where there is no space for KMC.

| Sex | Age | Quote | Comment |
| --- | --- | --- | --- |
| F |  | *“The greatest challenge I have got so far is inadequate oxygen concentrators. Sometimes when you initiate a baby on kmc, you find that we don’t have oxygen and there is no concentrator. So, when there is no oxygen, we have to use the minimum concentrators we have for all the babies which means we have to remove some babies from oxygen. That is one of the biggest problems”. (Respondent 2- AH)* |  |
| F |  | ***“****Ha, the CPAPs. We do not have enough. And when the mothers have C section or they are not well, they might not be able to give Kangaroo entirely” (Respondent 2 -CH).* |  |

**2.2 Sub-theme: Adequate space and beds**. Lack of sufficient space for KMC beds compared to the available number of babies and lack of privacy were described as a barrier to implementation of KMC at all the hospitals by the health care workers.

| Sex | Age | Quote | Comment |
| --- | --- | --- | --- |
| F |  | *“We have a challenge of space because sometimes babies qualify but we don’t have space where to put these mothers to practice kmc. The general patients have only four beds in the kmc room, and you may find you have like 10 babies for kmc. So, we have that challenge which leads us to the warmers. Space is a very big challenge” (Respondent 4 – AH).* | Inadequate space. Lack of KMC beds |
|  |  | *“We have to have beds for such mothers. We have to have a ward for Kangaroo; they should know that the preterms are in this ward of Kangaroo and the beds are there for the mothers. NICU babies don’t have beds and the only Kangaroo that is done there is on the chairs when they go to breast feed the babies. After feeding, they put the babies in Kangaroo. They don’t get to practice Kangaroo well, they just put the baby there for 30 minutes and they chase them from NICU. Then they will come back after four hours. But, if they knew that this was for preterms and the Kangaroo is theirs, and they cater for them when the mothers are there. Actually, we need beds” (Nurse- CH).* |  |
|  |  | *There is no ward for Kangaroo; no space for Kangaroo. If the space was there, then the mothers would start. (CH)* |  |
| F |  | *“Sometimes; inadequate space, low staffing because an unstable baby needs someone to monitor how the mother is doing it, status the baby. You find one nurse in those unstable babies alone where up to 50 mothers have come in. Sometimes you want a mother to do Kangaroo, but it is not applicable because of the big numbers. The mother might take-off the baby from oxygen while putting the baby in the chest because the nurse has told them to put babies in kangaroo. The baby might stop breathing since the nurse is working alone, she may not easily notice therefore the baby might need bagging. There is shortage of staff which makes monitoring hard because sometimes we are overwhelmed by the numbers, but we try our best” (Nurse- CH).* | KMC beds, low staffing levels |
|  |  | *“maybe it is the hospital setting, the space. If they discharge the mother this side, maybe they need another mother to take up this space. Yes, it is a good thing, but they really struggle to stay. You find that a mother who has delivered normally or by caesarean: If the hospital is too crowded, she may have to sleep on the floor which is not healthy enough for her; space is not there. So, they would even pressure us to tell us that nurse we want to go home they have discharged me from the other side why can’t you discharge me. Also, that is another challenge” (Respondent E- BH).* | Inadequate space and fewer KMC beds |

**2.3 Sub-theme: Inadequate number of healthcare workers**: Low staffing levels hinders the implementation of KMC before stabilization as the unstable babies need to be monitored and given other supportive treatment modalities. Almost all the hospitals expressed this as a challenge.

| Sex | Age | Quote | Comments |
| --- | --- | --- | --- |
|  |  | *“Sometimes; inadequate space, low staffing because an unstable baby needs someone to monitor how the mother is doing it, status the baby. You find one nurse in those unstable babies alone where up to 50 mothers have come in. Sometimes you want a mother to do Kangaroo, but it is not applicable because of the big numbers. The mother might take-off the baby from oxygen while putting the baby in the chest because the nurse has told them to put babies in kangaroo. The baby might stop breathing since the nurse is working alone, she may not easily notice therefore the baby might need bagging. There is shortage of staff which makes monitoring hard because sometimes we are overwhelmed by the numbers, but we try our best” (Nurse- CH).* |  |
|  |  | *“the staffing itself is a problem in implementing KMC. One staff is on duty, and you have 27 babies you can’t monitor all these babies and remember we the study nurses use these monitors. So we put the monitors on our babies. This unstable baby needs a monitor to help you at least monitor the baby as you do other work. But if the baby is not on a monitor, you are one on duty, you are giving treatment and they are bringing in admissions, so trust me if this mother puts this baby in kmc, you will find this baby dead” (Respondent 1- AH).* |  |

**2.4 Minimal hospital administration/leadership involvement**

There was reported minimal support from the hospital leadership towards implementation of KMC in general which hinders uptake of KMC prior to stabilisation.

| **Sex** | **Age** | **Quotes** | **Comments** |
| --- | --- | --- | --- |
|  |  | *“In my opinion, if I am to rate administrative involvement, it is almost 20% support and that is the provision of those four beds (interrupted by one respondent) and the oxygen which is in most cases running out every day and you have to chase (follow up) people for oxygen. So, we actually need people to push them to understand kmc so that they can help us. But it is not something that they have put to the front that it needs attention” (respondent 3 - AH).* | Involvement of hospital leadership as a facilitator |

**Barriers and facilitators of KMC before stabilization from the caregivers’ perspective**.

From the interview and FGD with the care givers on the facilitators and barriers of practicing KMC prior to stabilization, the emerging themes under facilitators of KMC were grouped under the WHO building blocks. These are: family involvement and support, medical devices and medicines, human resources for health and infrastructure. The emerging themes under the barriers of practicing KMC prior to stabilization were grouped under personal, cultural and environmental barriers.

**Facilitators of KMC prior to stabilization**

**1.0 Theme: Family involvement and support**

**1.1 Sub-theme: Financial support**

Family members and friends encouraged the caregivers to do KMC through financial support that helped the caregivers to pay for the associated upkeep costs. This is reflected in the quotes below.

| Sex | Age | Quotes | Comments |
| --- | --- | --- | --- |
| F | 30+ | *Where the man works, in the garage, they collected money for him like that for us to survive.” (IDI, F, AH)* |  |
| M | 20+ | *“Yes. By family support, I mean this. A family member might come and find you deep in thought, probably about lack of food at home. Then, they give you 50,000/-. At that time, the focus immediately goes back to the child. You send the 20,000/- to help at home and you remain with the 30,000/- to help at the hospital. I think you understand that; and at that point, you feel better.” (20+ years, IDI, M, CH)* |  |

**1.2 sub-theme: Psychosocial support**

Family members and friends helped the caregivers to sustain KMC through hospital visits, words of encouragement and in doing KMC as reflected in the quotes below.

| **Sex** | **Age** | **Quotes** | **Comments** |
| --- | --- | --- | --- |
| **F** | **30+** | *Even when I gave birth to this baby my brother in Mbale gave me hope because his brother- in-law also had a premature baby at eight months in Mbale hospital and he advised me to do what the health workers told me and everything will be okay.” (30+-year-old, F, IDI, DH)* |  |
| **FGD** |  | *Sometimes, I ask their father to help me put the babies in the chest when he comes back. And some days he does put them and you have to thank God when he wears the Kangaroo wrap.” (FGD, CG, DH)* |  |
| **M** | **-** | *If I had a caretaker helping my wife to wash, looking after the baby when the mother has taken a walk outside because we cannot leave the baby alone, they steal babies. So, the mother might be sleepy and go outside with the mat to take a nap and the caretaker stays with the baby. Even if the caretaker is here, it is me who is looking for what to eat and I bring for them. Even if I am in town and these two are here, it does not take away my responsibility...” (IDI, M, DH)* |  |
| **-** | **-** | *“You sit and pray to God because there is no standby generator when electricity goes off. You therefore worry about your child who is on oxygen for example my child is on oxygen but 2 days back, electricity went off. The only thing to do is to pray because it is not easy.” (FGD, CG, AH)* |  |

**1.3 Peer to peer support**

Being counselled by a person who has gone through same problem, helped many to cope with challenges associated with KMC as expressed in the quotes below.

| Sex | Age | Quotes | Comment |
| --- | --- | --- | --- |
| F | - | *During that time people would come in and they had brought their babies for review, and they would tell us their stories and encouraged us to be strong that also their babies were like that; that made them believe. (IDI, F, DH)* |  |

**2.0 Theme: Human resources for health**

Healthcare workers’ availability, ability to health educate caregivers, counselling and encouraging of caregivers, and their attitude motivated the caregivers to continue doing KMC for unstable neonates. This is reflected in the quotes below.

2.1 **Presence of healthcare workers**: Availability of healthcare workers who offer continuous counselling and health education.

| Sex | Age | Quote | Comments |
| --- | --- | --- | --- |
| F | 20+ | *The health workers treat the patients well. They tell you what to do and even when you lose hope, they keep motivating you. They encourage you to keep following a particular practice and that the results will be evident. When you continue doing it, you notice positive changes. That is what has kept me strong and also enabled me do Kangaroo.” (20+ years, IDI, F, CH)* |  |
| F | - | *The health workers were also supportive because even when they found you sleeping at night, they would wake you up. Do not sleep because you did not come here to sleep. They were indeed supportive.” (FGD, CG, DH); (NK, F).* |  |
| M | 20+ | *The truth is that the health workers taking care of these children have the experience. When they see you doing something the wrong way, they take their time in correcting and telling you how to do it. They do not mistreat you.” (20+ years, IDI, M, CH)* |  |
| F | - | *They have good care, it was my first time to come to this hospital but when they were bringing me here, I didn’t want to come because I know government hospitals don’t have care. But when I came here and went in kangaroo, I gave them credit for their care of these babies. I don’t know about the other wards but in this one they are really caring.” (IDI, F, BH)* |  |
| F |  | *You can go to some hospitals and they ignore you but when you are in kangaroo, it helps. There is a difference between the health workers because when I look at when I gave birth and when I joined kangaroo, the health workers in kangaroo were very caring. The kangaroo health workers for example would give us hot water in the morning, they would give us what to use to clean the baby’s umbilical cord. They would encourage us and make us hopeful, the others as long as their work was done you take care of yourself...” (IDI, F, DH)* |  |

2.2 **Healthcare worker knowledge and experience:** Health workers (HCW) advice to caregivers on how to perform KMC, general handling of preterm new-borns and the benefits of KMC encouraged them to continue doing KMC. Some HCW shared their stories of having preterm babies & how KMC helped them.

| Sex | Age | Quote | Comment |
| --- | --- | --- | --- |
| F |  | *The health workers made me get used. The health worker who came here told me that her last born was also a preterm baby and very small and she told me that is what she used to do. She told me that is what the doctors recommended her to do and encouraged me that baby will grow normally if I continue putting the baby in my chest daily. (CG, F, FGD, DH)* |  |
| M |  | *The health worker gave us a wrap and told us that it requires to put the baby in the chest on bare skin and tie the baby to get warmth, that because this baby is still very young it still need the mother’s warmth so when you put the baby in the chest it will be as if they are in the womb. My wife started putting the baby and she also told me that even if we go back home, we should continue putting the baby in the chest even I the husband I should help my wife to put the baby in the chest and we did, we would put the baby in the chest like for thirty minutes. (IDI, M, DH)* |  |
| F | _ | *The fact that I want my baby to be alive gives me hope and strength to do whatever I am told to do as long as it will help him. I have to follow what I am told so that I get what I want from it. I wouldn’t refuse to do what I have been told to do to enable my baby to add weight because I also want him to gain weight. I become happy when my baby who had a birth weight of 1.5kgs gains weight because I would lose hope if the baby was not gaining weight. When the baby who was 1.5kgs gains to 1.7kgs, I then see that there is hope. If I am to spend a second week here, you will find that the baby has gained more weight. So, in my opinion, KMC is helping the baby to gain weight. (IDI, F, CH)* |  |

2.3 **Healthcare worker attitude:** Positive attitude and encouragement to do KMC. The HCWs were very supportive in giving information to the caregivers and ensuring that caregivers practiced what they had been taught.

| Sex | Age | Quotes | Comments |
| --- | --- | --- | --- |
| F |  | *They treated us well, whenever we called them, they would come immediately to check on the baby. Maybe there are some people who can complain about them but for me, I have no complaints.” (IDI, F, BH)* |  |
|  |  | *“They tell you what to do. The truth is; the basawo are not bad because even when you have a complication, you see that they really care and they check on you all the time, ask if you need anything or if you have any question. If you have any challenge, you run to the musawo and you see that the musawo really cares to help you.” (FGD, CG, CH)* |  |
|  | 30+ | *The health workers constantly cared and checked on us; they always asked if I was performing KMC. They encouraged and directed us on how to perform the KMC.” (30+ years, IDI, M, AH)* |  |
|  |  | *“Even when one musawo has just told you to put the baby in KMC, another musawo will also come to tell you. They come all the time to tell you to put the baby in the chest.” (FGD, CG, CH)* |  |
|  |  | *“Even basing on how they took care of us and their attitude towards us, it would prompt one to give them money as a way of appreciation.” (IDI, M, BH)* |  |
| F | 20+ | *“The health workers treat the patients well. They tell you what to do and even when you lose hope, they keep motivating you. They encourage you to keep following a particular practice and that the results will be evident. When you continue doing it, you notice positive changes. That is what has kept me strong and also enabled me do Kangaroo.” (20+ years, IDI, F, CH)* |  |
|  |  | *“Tying the baby in the chest helped me a lot, secondly the kangaroo health workers were very caring.” (IDI, F, DH)* |  |

**3.0 Theme:** **Medical devices and medicines.**

**3.1 Sub-theme:** **Availability of medicine and timely treatment.** This enabled the caregivers to perform KMC with hope that the newborns will survive.

| Sex | Age | Quote | Comment |
| --- | --- | --- | --- |
| F |  | *They used to give the babies treatment, in one night they would get treatment like four times even during day the babies would receive treatment through cannulas all the time until we left when the babies had improved, they gave them good treatment.” (IDI, F, BH)* |  |
| F |  | *“The only medicine that we bought was used to remove mucus from the children. That is the only one that we have so far bought; the other is given to us.” (IDI, F, CH)* |  |
| F | 20+ | *“They receive treatment in the morning, afternoon, evening and in the night. The health workers constantly check on them. Even when they find us asleep, the health worker tells you to get up and ensure the child gets medicine. Then, you get up and they inject the child and go back to rest.” (20+ years, IDI, F, CH)* |  |
| F | 40+ | *“It was easy because we understood what they taught us and we didn’t forget how we were told to care for the babies even when the babies were off oxygen, the babies improved. The treatment of the babies for example when the babies couldn’t suckle well they were given glucose for a full week to get some energy. The medication given to babies through cannulas also made it easy.” (40+ years, IDI, F, DH)* |  |

**4.0 Theme: Infrastructure**

Available space and beds for practicing KMC on wards encouraged the caregivers to continue with KMC prior to stability. Caregivers reported privacy on the KMC ward as a motivator to continue with the practice as compared to the general wards that is too open and congested.

**4.1 Sub-theme: Adequate space and beds**.

| Sex | Age | Quotes | Comment |
| --- | --- | --- | --- |
| F |  | *“Every parent who is doing Kangaroo has their own bed. Nalongo (mother of two) was given two beds and she has enough space to do Kangaroo.” (IDI, F, CH)* |  |
| F |  | *“It doesn’t have a lot of people… this side we have enough space.” (FGD, CG, CH)* |  |

**Barriers of KMC prior to stabilization**

From the interview and the FGDs 2 overarching themes emerged, that is, personal and institutional factors.

1. **Theme: Personal barriers.**

**1.1 Sub-theme: Fear to hurt the newborn**. Caregivers feared hurting the unstable neonates who were still on oxygen and believe to be too sick and small to survive. This fear was further potentiated by the fear of disconnecting oxygen tubes and thus suffocating the neonate. Below are the related quotes.

| Sex | Age | Quote | Comments |
| --- | --- | --- | --- |
| F | 70+ | *“When the baby is on oxygen is not possible because the baby has machines in the nose, how can you carry the baby without those tubes coming out? They come out, I even saw it there when the tubes are long and connected to many things so how can you carry the baby? I even heard so many babies there died in the week we spent here.” (70+ years, IDI, F, DH)* |  |
| F | 40+ | *That’s where I stopped fearing* *and got used; I believed that this will work out. if I put much effort.” (40+ years, IDI, F, AH)* | Not understanding KMC effect |
| F |  | *“At first, I saw that it was a burden but after I believed that the preterm baby is my own and I want the baby to survive so I must do whatever they tell me...” (IDI, F, BH)* | Hopeless about the neonate’s survival |
| F |  | *“I think when they bring the baby when it needs oxygen, let them first put the baby on oxygen then when the baby has improved and breathing well then, the baby should be put in the mother’s chest better than putting the baby in the incubator. I don’t like the incubator.” (IDI, F, BH)* |  |
|  |  | *“I had fear especially when other baby had died so we still had that trauma, we still had that pain. So when they talked about removing the baby from the machine, when they put the baby out of the machine I saw like as if the life has gone out of her and I asked myself why the baby was put out of the machine. So there was fear because it was our first experience, our other daughter whom these twins follow was produced normally, she was okay so it was a new experience. Everything was for trying so we had fear in everything.” (IDI, M, DH)* | Loss of one twin. |
|  | 20+ | *“The truth is; I was afraid at first. My concern was that how could we do Kangaroo yet the child was still on oxygen. I wondered if the child would not die…When the baby is on oxygen, it sometimes changes positions. So, when you put the child in the chest, you might fall asleep. At this time, the child might change positions that could affect the supply of oxygen through the tube. That is the fear that I had.” (20+ years, IDI, M, CH)* |  |
|  |  | *“What scared me, these oxygen wires when you hold the baby, the wire pulls itself and looks like it’s going to move out, or move out and think that the baby will get poor air. So I wanted the baby to stay sleeping in position on its bed like where they were being put in the hospital. So what sacred me, was disturbing the baby to change the position from here to there and I was saying it’s better to leave the baby there but the way she was delicate she was told to hold the baby like this (in the chest) and cover with clothes.” (IDI, F, AH)* |  |
|  |  | *“I first thought the oxygen tubes would come out of the nose. The oxygen tubes were long up to the nose but I thought that the tubes might come out and the baby fails to breathe. You have to hold the baby with care and re-organise the oxygen tube before putting the baby in the chest to avoid hurting the baby’s nose.” (FGD, CG, AH)* |  |

**1.2 Sub-theme: Lack of knowledge on KMC.** Caregivers who heard about KMC for the first time did not understand how it would help their babies instead of the incubator. CG reported lack of training and miscommunication about KMC procedure. There was limited time by the healthcare workers to health educate the caregivers on KMC. This was further complicated by the different or mixed instructions from the health workers during different medical shifts.

| **Sex** | **Age** | **Quotes** | **Comments** |
| --- | --- | --- | --- |
| **F** | **-** | *Quote*  *“No, actually at first, I viewed it as a very difficult thing because it was the first time and I didn’t know kangaroo and it was the first time to hear about it. Just that when I had just arrived in the hospital, I met my co-wife who told me that her sister went through the same experience, and it was not easy. So you have to be determined.” (FGD, CG, CH)* |  |
| **F** | **40+** | *That’s where I stopped fearing* *and got used; I believed that this will work out. if I put much effort.” (45 years, IDI, F, AH)* |  |
| **F** | **-** | *“I thought it was going to be very difficult for me…Because I didn’t know it and had never used it.” (FGD, CG, AH)* |  |
|  |  | *“A doctor might come and tell you to give the baby 20 millilitres of breast milk but don’t breast feed. Another will come and complain as to why you are not breast feeding the baby and tell you to breast feed. They give different instructions so it would be better if it was one or two doctors giving instructions to avoid confusing us.” (FGD, CG, AH)* |  |
|  |  | *“Yes, if they come here at the facility and get trained on how to use it because we also first got trained on how to use the method before we started using it. Someone who is not trained on it cannot know how to use it.” (FGD, CG, DH)* |  |

**1.3** **Sub-theme: Financial constraints**. Many care givers lacked the finances to sustain them in the hospital while performing KMC on unstable babies. They required things like food, milk, pampers, wipers and money for upkeep.

| **Sex** | **Age** | **Quotes** | **Comments** |
| --- | --- | --- | --- |
|  | 15+ | *We had financial challenges and lack of necessities. They would send us for necessities like the drugs, pampers, syringes yet they all had to be bought. The necessities were many beyond our expectations and cost a lot. We thought we would be discharged earlier.” (15+ years, IDI, F, BH)* | Lack of medical supplies |
|  |  | *“We found a challenge in looking after us maybe because we didn’t have enough money yet we had to buy things to use. The fact that the child was premature, we didn’t have enough clothes for covering the baby. Even me I didn’t have things that bring money for eating. And the man has just developed and just married the woman and they don’t have enough money as they are staying in AH and renting a house and go to the village to farm. So the challenge we got in the hospital was finding things to use and to eat. We found a challenge and suffered in getting what to eat, and they hadn’t yet brought clothes because it was still early to give birth so that is what disturbed us.” (IDI, F, AH)* |  |
|  |  | *“The father of the baby had stopped giving us money for food, remember I was in ward eight for a whole month so when they told us to buy syrups for the baby, we called him because we didn’t have money anymore, but he told us that he didn’t have money anymore and that he was tired of the situation even the money for food we didn’t know where to get it from. When my sister went to Buwenge and got some food that is when we would get something to eat, that is the situation we were in. But after we got money and bought those syrups for the baby and the other things, they wrote for us when we were being discharged and I was confident that I would get the money to buy them when I went back home.” (IDI, F, BH)* |  |
|  | 15+ | *The mother got a loan from a certain SACCO she belongs to. She has not paid them yet.” (15+ years, IDI, F, BH)* |  |
|  |  | *“Some of us have husbands who refuse to look after premature babies and view them as babies that will not grow so they refuse to look after you in the hospital and tell you to take the baby home to die. So when you add the stress from home and that from the musawo who is supposed to encourage you, you cannot express breast milk and look after the baby.” (FGD, CG, AH* |  |

**1.4 Sub-theme: Lack of Physical support.** The lack of physical support or care giver to assist with KMC was reported by all the participants as a big barrier. Those with twins said that it was worse to perform KMC on two preterm babies and failure to have second care giver created preference treatment for one and the other twin received limited KMC.

| **Sex** | **Age** | **Quote** | **Comment** |
| --- | --- | --- | --- |
| **F** |  | *“Sometimes, I ask their father to help me put the babies in the chest when he comes back. And some days he does put them and you have to thank God when he wears the Kangaroo wrap.” (FGD, CG, DH)* |  |
| **M** |  | *“If I had a care taker helping my wife to wash, looking after the baby when the mother has taken a walk outside because we cannot leave the baby alone, they steal babies. So, the mother might be sleepy and go outside with the mat to take a nap and the care taker stays with the baby. Even if the care taker is here, it is me who is looking for what to eat and I bring for them. Even if I am in town and these two are here, it does not take away my responsibility...” (IDI, M, DH)* |  |

**1.5 Sub-theme: Tiredness.** KMC leads to tiredness and discomfort related to the positioning of the baby in KMC and maintaining it. Such inconveniences led to the participants putting the children on the bed and only performing KMC when they felt ready and comfortable.

| **Sex** | **age** | **Quotes** | **Comments** |
| --- | --- | --- | --- |
| **M** | **20+** | *This method is tiresome. For the mother who is doing this method, she needs someone to take over when she gets tired. Then, she also gets some rest… I think the words of encouragement are important. Also, the visits by the community members and parents of the mother of the child are key.” (20+ years, IDI, M, CH)* |  |
| **F** | **-** | *“In my opinion, it is much easier to do KMC at night than it is during day because there are lot of distractions during the day. For example, you may have just put the baby in the chest then the nurse comes to monitor the baby and tells you to put the baby down for monitoring. This may at times prevent the baby from sleeping again in that the baby who was being cuddled will not accept to sleep.” (IDI, F, CH)* |  |

**1.6 Sub-theme. Other cores.** Care givers had other competing activities to be done like washing clothes, buying food and drugs when never medicines were out of stock.

| **Sex** | **Age** | **Quote** | **comment** |
| --- | --- | --- | --- |
| **F** | **20+** | *When you have someone to help, you don’t need to remove the child from your chest. But when you don’t have someone to help, then you will remove the child every now and then. Sometimes you need to go to the bathroom or get food. Therefore, the child would not be able to get the necessary body warmth as they would have got if there was someone to help the mother.” (20+ years, IDI, F, CH)* |  |
| **F** | **20+** | *“If you are not used to it, you might fail to handle. When you have chores to do, you might fail to find time to do kangaroo. I used to get that challenge of just lying there and do kangaroo that’s why I only did kangaroo for just 3 months. I started to just cover the baby in warm blankets because I found it challenging and couldn’t handle anymore.” (20+ years, IDI, F, AH)* |  |
| **F** |  | *“If I am in hospital without a caretaker and I want to wash clothes, I want to go buy food I will have no one to stay with the baby so that makes it difficult.” (IDI, F, DH)* |  |
| **F** | **20+** | *When you have chores to do, you might fail to find time to do kangaroo. I used to get that challenge of just lying there and do kangaroo that’s why I only did kangaroo for just 3 months. I started to just cover the baby in warm blankets because I found it challenging and couldn’t handle anymore.” (20+ years, IDI, F, AH)* |  |

**1.7 Sub-theme: Maternal morbidity.**

Many reported that C sections were a hindrance and that the mothers failed to do KMC because they were in great pain.

| **Sex** | **Age** | **Quote** | **comments** |
| --- | --- | --- | --- |
|  |  | *“However, after that I fell sick, I could not walk and never wanted to lay down or sit. I had swollen legs and I found difficulties laying or sitting down. When I went to the pressure room, I was told to avoid unnecessary movements but at the same time; I was supposed to always go to the room where the babies were to breastfeed them or express milk for them and my breasts did not have milk. I also did not have appetite to eat or drink. I generally found difficulty in almost everything.” (FGD, CG, DH)* |  |
|  |  | *“God helped me and I gave birth normally. However, my biggest challenge was that my caretaker was an elderly woman who could not take good care of the babies. I had to always walk to check on the babies and I also sleep with the babies. Because of this, my legs and chest got swollen which made things difficult for me. I was told to get money to buy a certain drug to be injected for the chest to go back to the normal size. I had swellings in my armpits yet I had to express milk for the babies. The babies had not started breastfeeding so I was supposed to express milk them. It was difficulty for because I was going through a lot of pain and the caretaker was elderly and could not support in most things.” (FGD, CG, DH)* |  |
| **F** | **20+** | *I experience back ache when I put the child in my chest when in one position facing up. I also get chest pain as a result of putting the child in the chest for long.” (20+ years, IDI, F, CH)* |  |
| **F** |  | *“After the operation, the baby was put in a separate room as I remained on the sickbed in the ward. I was forced to always check on the baby where he was. This was very difficult for me to always go to check on the child because I would feel a lot of pain to walk and set on the chair since I had gone through an operation.” (FGD, CG, DH)* |  |

**1.8 Sub-theme: Caregiver negative attitude.** Caregivers’ negative perception towards the survival of the preterm neonates hindered the ability to do KMC.

| Sex | Age | Quotes | Comments |
| --- | --- | --- | --- |
| F | 20+ | *They tell us parents what to do but we don’t care. You tell a mother to put the baby in kangaroo and they think it is not good that maybe you’re wasting their time yet your helping their baby even the mother herself, because if the baby dies then the mother will say that it is the health worker who has led to the baby dying yet the health worker is telling you to put the baby in kangaroo.” (20+ years, IDI, F, DH)* |  |
| F | - | *“It comes as a result of bad attitude, which includes big headedness, poor understanding and carelessness. Let’s take an example, if the health worker tells you that a mother should bathe 2-3 times while performing KMC, you should. Some of the adults have their diseases but some are unaware of them. But the health worker is telling us to be hygienic so that the babies don’t contract anything from the adults. But due to the big headedness, poor understanding, and carelessness, when the mothers leave the health worker’s presence, they undermine the message of the health workers because the babies don’t belong to the health workers. Even if such go back home, they might spend two days without bathing though they continue putting the child in their chest.” (IDI, F, CH* |  |

**1.9 Sub-theme: Long distance for follow up visits.** When they were supposed to go back to the hospitals for follow up visits, they found it as a big challenge because it involved a lot of costs in terms of transportation.

| Sex | Age | Quotes | Comments |
| --- | --- | --- | --- |
| F |  | *“In addition to that; there are children who come from very far in the villages. So I am suggesting that since some of us come from very far, you (health workers) can inquire about the condition of the children from the parents and even if health workers do not reach there, they can make phone calls to follow up on these children because they became your (health workers’) children. Sometimes they might be in bad conditions. That is what I am suggesting.” (FGD, CG, CH)* |  |
| F | 20+ | *“In my point of view, there are women who give birth and are unable to reach the hospital. Sometimes it is due to the big distance yet they require this service but cannot do so. I am suggesting that if possible, you do an outreach program in those rural areas that do not have this service. Then, you go to a community and teach them about the Kangaroo Method, so as to increase their awareness. Right now, there are very few people who know about this method. It would be good if you went around teaching people about the method.” (20+ years, IDI, F, CH)* |  |
|  |  |  |  |

**2.0 Theme: Cultural barriers.**

**2.1 Sub-theme: Cultural beliefs.** Cultural beliefs such as an infant cannot see you naked and performing KMC in the presence of **t**he in-laws. Some cultures do not accept women to expose their bodies in the presence of such persons and viewed it as a barrier to performing KMC.

| **Sex** | **age** | **Quotes** | **Comment** |
| --- | --- | --- | --- |
| **F** |  | *The grandmother to the baby cannot undress and do kangaroo in the presence of the baby’s father, you have to tell your son-in-law to go out of the room then you can do kangaroo and when I finish I take the baby out of the chest then I call him to come and see the baby. I don’t even think that we can change that because we also found it there…My daughter has to continue with the Kangaroo. What I told you earlier was related to my case. Of course, she would not be doing it in front of her father-in-law. She would be doing in the bedroom. By the way, why would the father-in-law be staying at their place!...Even the father-in-law cannot look at the daughter-in-law when they are bare chested.” (IDI, F, CH)* |  |
| **M** |  | *They can’t imagine us men putting our babies in the chest for a while; they usually think of other methods like using a hot charcoal stove or putting jerry cans filled with hot water around the baby.” (FGD, CG, BH)* |  |
|  |  | *Also when people say that a baby born at eight months doesn’t survive, it can scare the mother and make her lose hope.” (30+ years, IDI, F, DH)* |  |

**2.2 Sub-theme: Cultural practices.** Care givers preferred the other alternatives of warming up small newborns such as charcoal stove to warm the room and the bed.

| **Sex** | **Age** | **Quotes** | **Comment** |
| --- | --- | --- | --- |
| **F** | **-** | *Some of them wonder how we are able to give them the warmth they deserve; they usually ask if we are going to light a charcoal stove that will emit some heat. They usually laugh whenever we talk of kangaroo mother care” (FGD, CG, BH)* |  |
| **F** | **20+** | *“Yes, like I told you that they had told me doing kangaroo will not work. They told me to get jerry cans and put warm water in them, and put them around the baby and cover him with 4 blankets.” (20+ years, IDI, F, AH)* |  |
| **F** | **-** | *“They said that they did not expect the baby to survive but my sister told them that she also gave birth to a preterm baby and she also took the baby to hospital and the baby survived.” (IDI, F, DH)* |  |
| **F** | **20+** | *“In my view, this one (KMC) is much better compared to their methods. The people in the community give themselves advice and sometimes refuse to take the children to the hospital. They opt to cover the children. Sometimes, they share bad advice that in turn affects the babies.” (20+ years, IDI, F, CH)* |  |
| **-** | **-** | *“There are those you may explain to and they understand, some think that we are just wasting our time by doing Kangaroo. They wonder how a child can develop when placed in the chest. They wonder and ask if its practical for a child to grow under such circumstances- children do not develop when placed in the chest.” (FGD, CG, BH)* |  |

**3.0 Theme: Environmental barriers.**

**3.1 Sub-theme: Fear of damaging medical equipment.** Fear of damaging medical equipment was reported as a big barrier by many of the care givers.

| Sex | Age | Quotes | Comments |
| --- | --- | --- | --- |
| - | - | *Another issue musawo; remember some of us come from the villages and do not know the machines (oxygen tubes and CPAP) and have never seen them. We develop fear of the machine since it is the one helping my baby to breathe, what if you touch it yet you do not know how to put it right and instead you be the one to kill your own baby. So it is necessary for us who come from the villages to be taught how to put the baby’s oxygen tube right.” (FGD, CG, CH)* |  |
| F | - | *“The way those machines beep while making noise; I don’t want noise. I want to do kangaroo in a quiet place, but the machines beeped all the time.” (IDI, F, BH* |  |

**3.2. Sub-theme: Poor hygiene**. The unclean environment of the hospitals and the washrooms was another hindrance reported. The poor state of the toilets and the inability of the hospital to clean the gowns worn by the KMC mothers was reported as a barrier.

| Sex | Age | Quote | Comment |
| --- | --- | --- | --- |
|  |  | *Quote*  *“Uncleanliness in your environment for example when you enter a dirty place or you don’t wash hands but you come and touch the baby.” (FGD, CG, AH)*  *“* |  |
|  |  | *Also, if a cloth falls down, you are not supposed to put it back on the baby immediately because there may be germs where the cloth fell that may affect the baby. For example, this hospital is very dirty, you may even fear stepping on the floor because the floor is always dirty.” (FGD, CG, DH)* |  |

**3.3 sub-theme: Inadequate and faulty equipment:** Limited number of equipment leading to sharing of incubators and patient monitors which made the caregiver worry about their newborns getting infected. Also, the faulty incubators and warmers which would overheat sometimes made some caregivers worry about the hospital environment and wanted to go home. This would happen whenever the neonates were returned to the incubators. Also mentioned was the occasional overcrowding of the KMC room.

| Sex | Age | Quotes | Comment |
| --- | --- | --- | --- |
| F | - | *“I personally suggest that they should buy more machines used in the incubator because the equipment available are inadequate for the number of patients at the facility. For example, my baby was put something on the leg for about one month. Remember there were new babies who needed it yet they were not enough for the patients. Sometimes, they would remove it from my baby and put it on another baby, then bring it back after some time. Therefore, they should increase on the number of equipment at the hospital.” (FGD, CG, DH)* |  |

**3.4 Sub-theme: Perceived negative attitude of HCW:** The community believed that the health workers don’t care about patients and that there was no medicine in the hospitals most of the time. With such concerns, the community wondered how the health facilities would be able to cater for the preterm babies if they could not cater for the older babies. Some participants reported the poor attitudes of the health care workers and their numbers assigned to the neonatal or KMC ward were few. The care takers said that sometimes when they needed the nurses or doctors, they were not available; that there were very few especially during the night shift. Others said a number of the health workers were rude; they shouted at them so much every time they didn’t do what was expected. They added that even when the health workers didn’t find the care giver with the baby at the time of administering the medication, they did not come back to do so until the next day. They said that such unprofessional behaviour increased on the stress and worries the care givers.

| **Sex** | **Age** | **quotes** | **Comments** |
| --- | --- | --- | --- |
|  |  | *To make it better okay the nurses should change, they reach a point where they shout at us yet even us we sometimes we are not yet used to the babies and still scared. Nurses should also stop being tough. Some would say only those who have tied the babies in the chest will be registered in the file. like us we were in this room, but the nurse would not bother asking us to take the babies to attend to them. I myself experienced it when the nurse didn’t ask and instead got out from the other door and our babies were not attended to yet the nurse signed in the file. Even when you explain to another nurse that the baby was not checked, they cannot agree because the file shows something else, they should change their ways.” (IDI, F, DH)* |  |
|  |  | *“We request the basawo working there to stop acting like army people. You come stressed about the condition of your baby on oxygen and worried that the baby might die. You inquire from the musawo on what to do since you don’t know but instead of telling you what to do, he or she responds rudely or abuses you. You feel like the musawo adds on your stress since you are already stressed and still tells you to express breast milk for the baby at that same time. How do I express breast milk for the baby when I am stressed!” (FGD, CG, AH)* |  |
| **F** | **20+** | *“Sometimes you find when the child’s health has changed. You talk to the health workers about it and they tell you that they are going to address the issue. Then, when you go back at the designated time, you find that they didn’t do it.” (20+ years, IDI, F, CH)* |  |
|  |  | *“If the health workers come to the wards to treat the babies and do not find the medicine they asked you to buy or do not find you where the baby is, they would not treat your baby. Sometimes, they would inject the baby with the same medicine over time which made me think they were not doing the right thing. By the time you come back, your colleagues simply tell you the health worker has left and sometimes it is late in the night. However, this would be caused by lack of medicine or the money to buy the medicine. When the time to leave the ward reaches, the health workers leave the ward and your baby misses out on getting treatment.” (FGD, CG, DH)* |  |

**3.5 Sub-theme: Lack of medicines and supplies**

Some of the required medicines for the care of sick and small new-borns was not readily available in the facilities and caregivers were asked to go out of the hospital to buy. Caregivers would sometimes leave the newborns on the bed and go to the pharmacies outside the hospital.

| **Sex** | **Age** | **Quote** | **Comments** |
| --- | --- | --- | --- |
| **F** | **-** | *“If the health workers come to the wards to treat the babies and do not find the medicine they asked you to buy or do not find you where the baby is, they would not treat your baby…By the time you come back, your colleagues simply tell you the health worker has left and sometimes it is late in the night. However, this would be caused by lack of medicine or the money to buy the medicine. When the time to leave the ward reaches, the health workers leave the ward and your baby misses out on getting treatment.” (FGD, CG, DH)* |  |
| **F** | **20+** | *“The hospital usually provides the medicine but sometimes it is not available and you have to buy it. For example, there was a woman who had bought the same medicine that I had been provided with at the hospital.” (20+ years, IDI, F, AH)* |  |
| **F** | **-** | *“Musawo might send you for medicine and you fail to get it. However, when you go back to inform the musawo, he asks for money to get the medicine for you which leaves you wondering where he is going to get the medicine from at that time.” (FGD, CG, AH)F* |  |
