# Supplementary material for: Kangaroo mother care prior to clinical stabilisation: Implementation barriers and facilitators reported by caregivers and healthcare providers in Uganda
Source: PLOS Glob Public Health. 2024 Jul 31;4(7):e0002856. doi: 10.1371/journal.pgph.0002856 (PMC11290675; doi:10.1371/journal.pgph.0002856)
Supplement: Attachment — Submitted filename: Responses to reviewers.docx [file pgph.0002856.s003.docx]

**London School of Hygiene & Tropical Medicine**

(University of London)

Faculty of Infectious and Tropical Diseases

Keppel Street, London WC1E 7HT

28^th^ March 2024

Dear Dr Anteneh Asefa Mekonnen and the PLOS Global Public Health editorial team,

**Re: PGPH-D-23-02561**

**Title: *Kangaroo Mother Care prior to clinical stabilisation: Implementation barriers and facilitators reported by caregivers and healthcare providers in Uganda.***

Thank you for these thoughtful comments and insights from the two reviewers. We are delighted to see some positive feedback. We have taken all the comments very seriously and responded to each of the reviewers’ comments one-by-one, making revisions and improvements in the manuscript.

Please let us know if more information is required.

Yours sincerely,

Victor Spector Tumukunde

| **Reviewer 1 Comments:** | **Response** |
| --- | --- |
| Strong research design and well written, and it is coherent. The research used a qualitative inquiry and presented insight from the perspective of families/caregivers, health care workers and facility mangers and report important insight to barriers and facilitators to deliver kangaroo mother care in medically unstable state. I suggest the following revisions:   - In methods authors could describe how the adequacy of sample size was determined. It is not clear how the adequacy of reported sample size is determined. | Thank you for the positive feedback on research design and paper writing.  The adequacy of sample size was based on saturation. Data were collected until the team could not identify new responses. We have made this clearer by adding changes to the text in lines 145 & 146. |
| In the result and discussion section more insight on highlighting financing issues-results from both families and health facility managers reported as important barriers or enablers. For example, inadequate number of staffing, space, equipment could be due to low budget allocation. The opportunity cost to families coupled with the fact that families must buy medicine, food and other supplies is leading to increasing out of pocket expenses leading to catastrophic family expenses (as reported by other authors).   - The authors need to further examine the data. - If the authors agree with my suggestion above, I suggest revising the conclusion and recommendation section to highlight adequate financing beyond infrastructure remodelling. | Thank you for the comment, and we agree with you on the importance of low budgetary allocation likely to be a driver for health systems barriers, notably space and human resources. This was mentioned in the discussion in lines 508 to 510. We have added more text to underline the finance gaps in the conclusion and recommendations, as you suggest. We have also noted that a national neonatal investment case analysis is in process.  However, we are unable to comment on the catastrophic family expenditure given that this study did not go further to explore this issue, although non-direct costs of transport and food plus expenditures on medicines and supplies were mentioned.  In addition, we would like to note that the economic evaluation of the OMWaNA trial assessed the incremental costs and cost-effectiveness of KMC before stabilisation relative to standard care from a societal perspective, including opportunity costs to households. These findings will be reported in a forthcoming manuscript in *The Lancet*:  Tumukunde V, Medvedev MM, Tann CJ, Mambule I, Pitt C, Opondo C, et al. Effectiveness of kangaroo mother care before clinical stabilisation versus standard care among low-birthweight neonates at five hospitals in Uganda (OMWaNA): a parallel-group, individually randomised, controlled trial and economic evaluation. Lancet. 2024 Jan; in press. |
| **Reviewer #2 Comments** |  |
| ***Good study.***   - The abstract as it will be available separately should have no abbreviations such as IDI, KIIs | Thank you. We have provided the full spelling of all abbreviations the first time they were used in lines 25, 32, 34, 35 and 36 in the abstract and in the tracked changed version. |
| - You included the research assistants in the focus group which is concerning. They were part on the implementing team. | Thank you. The research assistants who were included in the focus group were the healthcare workers who had been trained in KMC prior to stability, while the healthcare workers at the trial hospitals were not trained in KMC prior to stability. The purpose of this was to get their different views given the differences in the knowledge of the intervention. |
| - Not sure how the reader is expected to interpret focus group discussions separately from the IDI. For a non-social scientist reading the study, this should be clarified in the methods. | We have explained the value, and complementarity, of the two methods in the methods section – as suggested, please see lines 154-157 in the tracked changed version:  `Individual interviews allowed respondents to share personal experiences, views and stories, while the group discussions offered the opportunity for small groups of respondents to share ideas, serving to stimulate discussion amongst those present.’ |
| - Table 1- first time we see a control group and not sure how the control group comments were dealt with. Were they analyzed separately, were they included because you needed the numbers or some other explanation. | Thank you, and we apologise if this was unclear. In the trial, mothers/caregivers in the intervention arm practiced KMC while their newborns were clinically unstable, while mothers/caregivers in the control arm practiced KMC after their newborns met pre-defined criteria for clinical stability. Our study design was to interview caregivers in both arms. This has been clarified in line 139 of the tracked changed version. |
| - In the results for example line 191 to 202 even though you are trying to tell reader what the Healthcare worker said, the quotes are from parents/mothers. It is difficult to tease out the healthcare worker concerns and family concerns Medical supplies again mothers quotes. | Thank you, and we apologise if this was unclear. The positive attributes of family support were mentioned by both the healthcare workers and caregivers. In the manuscript we included the quotes from the caregivers (lines 213-234). Also added a quote from the healthcare worker in line 235-238 of the tracked changed version.  For medical supplies and devices, we also captured both the views of the healthcare workers and the caregivers. Hower quotes from the healthcare workers are included as seen in line 369, 381 and 385 of the tracked changed version. |
| - Table 2 is good, but you do not flow the quotes according to your table of themes. | Thank you for this observation. Adjustment of the flow has been made in table 2. |
| - In the discussion you indicate cultural beliefs, but I may have missed, I did not see these in the IDI. The story is compelling, and you have a lot of good quotes, however it becomes quite long, and the message can be given in a shorter version that people will more likely read. | Thank you, and we apologise that this was unclear.  The cultural beliefs are mentioned in lines 257-258, 272-276under the family support. |
