# Supplementary material for: Kangaroo mother care prior to clinical stabilisation: Implementation barriers and facilitators reported by caregivers and healthcare providers in Uganda
Source: PLOS Glob Public Health. 2024 Jul 31;4(7):e0002856. doi: 10.1371/journal.pgph.0002856 (PMC11290675; doi:10.1371/journal.pgph.0002856)
Supplement: Attachment — Submitted filename: Responses to editorial comments.docx [file pgph.0002856.s004.docx]

**London School of Hygiene & Tropical Medicine**

(University of London)

Faculty of Infectious and Tropical Diseases

Keppel Street, London WC1E 7HT

6^th^ June 2024

Dear Dr Anteneh Asefa Mekonnen and the PLOS Global Public Health editorial team,

**Re: PGPH-D-23-02561**

**Title: *Kangaroo Mother Care prior to clinical stabilisation: Implementation barriers and facilitators reported by caregivers and healthcare providers in Uganda.***

Thank you for these thoughtful comments and we are delighted to see some positive feedback. We have taken all the comments very seriously and responded to each one-by-one, making revisions and improvements in the manuscript.

Please let us know if more information is required.

Yours sincerely,

Victor Spector Tumukunde

| **Editorial 1 Comments:** | **Response** |
| --- | --- |
| Please make it clear in the Methods that the presence of researchers as participants enhances the credibility and trustworthiness of your study, given its qualitative nature. | Thank you for the guidance, this has been addressed in lines 132-135 of the tracked manuscript. The statement is as follows: ***Research assistants had been trained in KMC prior to stability and were familiar with the practice which led to more candid and informed responses as opposed to responses referring to conventional KMC***. |
| Please also include a sentence or two about the limitations of potential biases that may have been introduced by having the research assistants as participants. | Thank you for the comments. We have revised the section of the limitation under lines 546-547 and this statement has been included; The ***involvement of research assistants as participants in the key informant interviews could have unintentionally generated biased responses.*** |
| Please review your reference list to ensure that it is complete and correct. If you have cited papers that have been retracted, please include the rationale for doing so in the manuscript text or remove these references and replace them with relevant current references. Any changes to the reference list should be mentioned in the rebuttal letter that accompanies your revised manuscript. If you need to cite a retracted article, indicate the article’s retracted status in the References list and also include a citation and full reference for the retraction notice. | Thank you for the guidance. All the references have been reviewed the list is complete and correct, and none has been retracted. |
